# Evaluation of IGF1/IGFBP3 Molar Ratio as an Effective Tool for Assessing the Safety of Growth Hormone Therapy in Small-for-gestational-age, Growth Hormone-Deficient and Prader-Willi Children

**DOI:** 10.4274/jcrpe.galenos.2019.2018.0277

**Published:** 2019-09-03

**Authors:** Meriem Gaddas, Laurence Périn, Yves Le Bouc

**Affiliations:** 1 University of Sousse, Faculty of Medicine ‘Ibn el Jazzar’, Department of Physiology and Functional Explorations, Sousse, Tunisia; 2 Trousseau Hospital, Assistance Publique-Hôpitaux de Paris, Department of Pediatric Endocrinology, Paris, France; 3 Sorbonne Université, INSERM, Centre de Recherche St-Antoine UMR S938, Assistance Publique-Hôpitaux de Paris, Trousseau Hospital, Department of Pediatric Endocrinology, Paris, France

**Keywords:** GH therapy, IGF1/IGFBP3 molar ratio, growth hormone deficiency, small for gestational age, Prader-Willi syndrome

## Abstract

**Objective:**

IGF1 concentration is the most widely used parameter for the monitoring and therapeutic adaptation of recombinant human growth hormone (rGH) treatment. However, more than half the variation of the therapeutic response is accounted for by variability in the serum concentrations of IGF1 and IGFBP3. We therefore compared the use of IGF1/IGFBP3 molar ratio with that of IGF1 concentration alone.

**Methods:**

We selected 92 children on rGH for this study and assigned them to three groups on the basis of growth deficiency etiology: small for gestational age (SGA), GH deficiency (GHD) and Prader-Willi syndrome (PWS). Plasma IGF1 and IGFBP3 concentrations and their molar ratio were determined.

**Results:**

Before rGH treatment, mean IGF1/IGFBP3 molar ratio in the SGA, GHD and PWS groups was 0.14±0.04, 0.07±0.01 and 0.12±0.02, respectively. After the initiation of rGH treatment, these averages were 0.19±0.07, 0.20±0.08 and 0.19±0.09, within the normal range for most children, even at puberty and despite some significant increases in serum IGF1 levels.

**Conclusion:**

We consider IGF1/IGFBP3 molar ratio to be a useful additional parameter for assessing therapeutic safety in patients on rGH, and for maintaning the values within the normal range for age and pubertal stage.

**What is already known on this topic?**

Growth hormone (GH) therapy is widely used, but concerns have been raised that it might increase cancer and cardiovascular risks.

**What this study adds?**

This study provides support for the use of IGF1/IGFBP3 molar ratio as a tool for assessing the safety of the therapeutic adaptation of GH therapy in children.

## Introduction

IGF1 serum concentration remains the most widely used parameter for the monitoring and adjustment of recombinant human growth hormone (rGH) treatment (1). However, more than 58% of the variation in the therapeutic response to rGH over the first year of treatment in children can be explained by the variability of serum concentrations of IGF1 and IGFBP3 (2).

In current practice, due to technical difficulties,total IGF1 concentration is usually measured without determination of free IGF1 levels (2). Free IGF1 can be assumed as the bioactive form, but it is in equilibrium with bound IGF1 engaged in large and small complexes, according to the mass action law; almost 99% of the serum IGF1 is engaged in such large and small complexes (1).

It is therefore impossible to determine tissue bioavailability and, thus, therapeutic efficacy or safety of IGF1 from IGF1 or IGFBP3 assays alone. Furthermore, a lack of correlation between daily GH secretion and total IGF1 concentration has been reported in some cases (1). This situation may be due to the presence of various polymorphisms affecting sensitivity to GH, which may differ for IGF1 and IGFBP3. It should also be noted that IGF1 concentrations vary considerably among individuals (3,4).

Finally, the dynamics of serum concentrations of IGF1 and its carrier protein, IGFBP3, remain unclear in patients on rGH treatment, and conflicting results have often been obtained (5). Moreover, high IGF1 concentrations have been implicated in cancer, whereas IGFBP3 has a protective effect (6). We therefore believe that determining the bioavailability of IGF1 by calculating the IGF1/IGFBP3 molar ratio would provide more information about the safety of rGH treatment than the use of total IGF1 assays alone.

## Methods

This retrospective study was performed at the Hormonology and Functional Endocrine Explorations Laboratory of Armand Trousseau Hospital in Paris. We selected, from our database, 92 children on rGH treatment followed at our outpatient clinics. These children were assigned to three groups on the basis of the etiology of their growth deficiency: 20 children who were small for gestational age (SGA group), 61 children with GH deficiency (GHD group), and 11 children with Prader-Willi syndrome (PWS group) (see Table 1A). Retrospective study of the patients’ files covered the period 2011-2017 and the clinical and biological data under treatment corresponded to the period March 2016-March 2017. The duration of the treatments and the follow-up of the patients varied from two to seven years.

The doses of rGH administered were as follows: 34.96±14.35 µg/kg/day for the SGA group, 25.56±10.01 µg/kg/day for the GHD group and 21.67±8.45 µg/kg/day for the PWS group. No adverse events attributable to rGH were reported in these children during follow-up.

Clinical [etiological diagnosis, height, weight, body mass index (BMI), rGH dose] and biological (IGF1, IGFBP3, insulin, fasting glycemia, lipid status, HbA1c) data were collected by consulting the patient’s medical records. Clinical parameters (height, weight and BMI) were standardized relative to the corresponding national reference ranges (7). IGF1 and IGFBP3 are expressed as Z-standard deviation (SD) scores (SDS) adjusted for age, sex and pubertal stage. Baseline clinical and biological characteristics were obtained for each of the etiological groups (Table 1A). National ethics and confidentiality rules were respected, in accordance with the legislation in force for retrospective studies of cohort files.

The IGF1 and IGFBP3 assays were performed with the IDS-Isys system (Immunodiagnostic Systems, 153 Avenue d’Italie, 75013 Paris, France), in an automated procedure based on ELISA with detection by chemiluminescence. The IDS-Isys device was calibrated according to the new World Health Organization international standard for IGF1 NIBSC 02/254, in accordance with the recommendations of the Growth Hormone Research Society and the International IGF Research Society (8,9,10,11).

Serum IGF1 and IGFBP3 concentrations were interpreted by comparison with the reference intervals established specifically for this iSYS device by Bidlingmaier et al (10) for IGF1 and by Friedrich et al (11) for IGFBP3.

The IGF1/IGFBP3 molar ratio was calculated according to the formula as previously described (11,12): [IGF1 (ng/mL) x 0.13] / [IGFBP-3 (ng/mL) x 0.035].

The reference values used for the interpretation of IGF1/IGFBP3 molar ratios were based on the data collected in our pediatric functional endocrine exploration department at Trousseau Hospital (see Table 1B). These data come from a control population made up of children who came to consult for follow-up of unrelated pathologies: moderate well-balanced asthma follow-up under inhaled corticosteroid, isolated hypospadias or testicular ectopy, moderate growth retardation of around -1 SDS and treated hypothyroid children stably euthyroid. These data were obtained from control children of different ages and pubertal stages and were comparable, as described by Friedrich et al (11), for IGF1/IGFBP3 molar ratio determinations.

The study design (retrospective analysis of the data) was approved by the Ethical Committee of Trousseau Hospital without an approval number. Informed consent has been obtained from the parents after full explanation of the purpose and nature of all procedures used.

### Statistical Analysis

Prism 6 Software (GraphPad Software, La Jolla, CA, USA) was used for all statistical analyses. ANOVA was performed to compare quantitative and qualitative variables between groups. A paired t-test was performed to compare qualitative variables between groups. Differences were considered significant if the p value was <0.05. A correlation analysis (Spearman) was carried out between the individual values of the three ratios and those of quantitative parameters.

## Results

**Figure 1:**
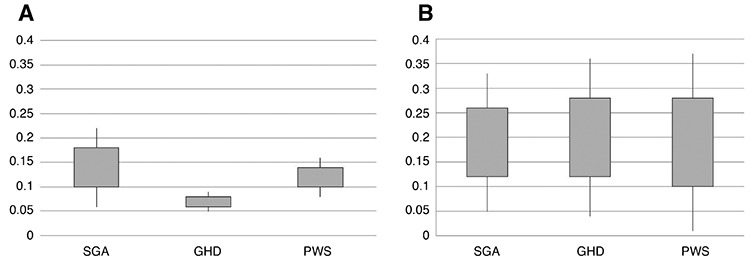
Distribution of IGF1/IGFBP3 ratio in the three groups before (A) and after (B) recombinant human growth hormone treatment. Rectangles represent values between +1 and -1 standard deviation scores (SDS) and bars represent SDS values SGA: small for gestational age, GHD: growth hormone deficiency, PWS: Prader-Willi syndrome

Before rGH treatment, the mean±SD molar ratio was 0.14±0.04 (range: 0.09-0.23) in the SGA group, 0.07±0.01 (range: 0.06-0.08) in the GHD group and 0.12±0.02 (range: 0.10-0.14) in the PWS group (Figure 1A).

The IGF1/IGFBP3 molar ratio values of the various groups in this study improved on rGH treatment, reaching values in the normal range for healthy children of the same age (Table 1A, 1B). Mean±SD IGF1/IGFBP3 molar ratio on rGH treatment was 0.19±0.07 (range: 0.12-0.35) for the SGA group, 0.20±0.08 (range 0.04-0.36) for the GHD group and 0.19±0.09 (range: 0.04-0.32) for the PWS group (Figure 1B). There was a positive correlation between the individual dose values of rGH and the individual values of the whole ratio (p=0.03). However the correlation between low-normal and high ratio groups and growth increments was not significant (p=0.15), probably due to the heterogeneity of age and duration of treatment.

**Figure 2:**
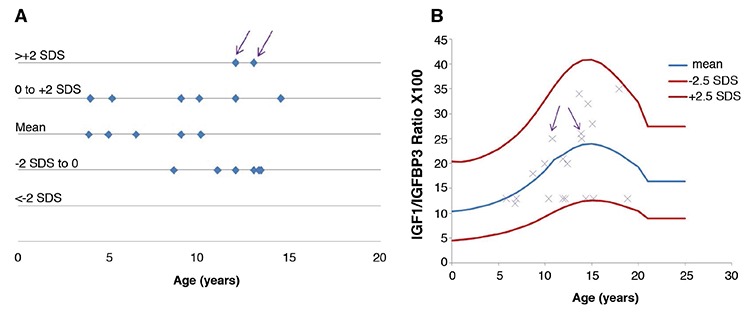
Distribution of IGF1 concentration (A) and IGF1/IGFBP3 molar ratio (B), in small for gestational age (SGA) children during growth hormone treatment. The IGF1 values have been distributed according to the standard deviation scores (SDS) intervals established by Bidlingmaier et al (10). In the SGA group, two children had IGF1 concentrations >+2 SDS, but IGF1/IGFBP3 molar ratios in the normal range (arrows)

In the SGA group (Figure 2), two children with high serum IGF1 concentrations (>+2 SDS) had normal IGF1/IGFBP3 molar ratios, because their IGFBP3 levels were also high (>+2 SDS).

**Figure 3:**
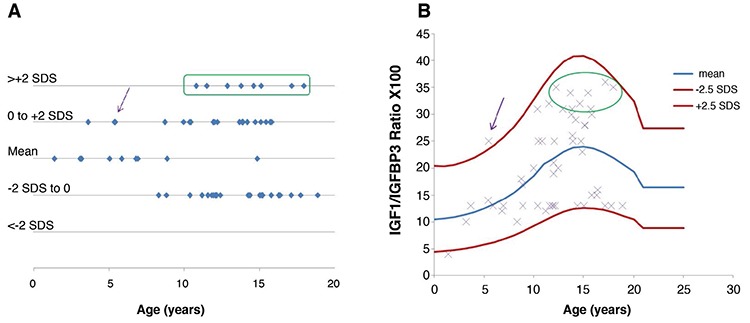
Distribution of IGF1 (A) and IGF1/IGFBP3 molar ratio (B), in children with growth hormone deficiency (GHD) during GH treatment. In the GHD group, eight children had IGF1 concentrations >+2 SDS (circled cases), but IGF1/IGFBP3 molar ratios in the reference range. Conversely, one five-year-old child had an IGF1 concentrations in the reference range but a very high molar ratio due to a very low IGFBP3 concentration (<-2 SDS; arrow)

In the GHD group, an analysis of IGF1 concentration according to age and pubertal stage after treatment revealed that 13% of children had IGF1 concentrations >+2 SDS, particularly during the pubertal period. However, when IGFBP3 levels were also taken into account, the IGF1/IGFBP3 molar ratios of these children were found to be in the normal range for age and pubertal stage (Figure 3, Table 1B). We also found one five-year-old child with a normal IGF1 concentration but a very high molar ratio (>+2 SDS), because of a very low IGFBP3 concentration (<-2 SDS), requiring therapeutic adaptation (Figure 3).

**Figure 4:**
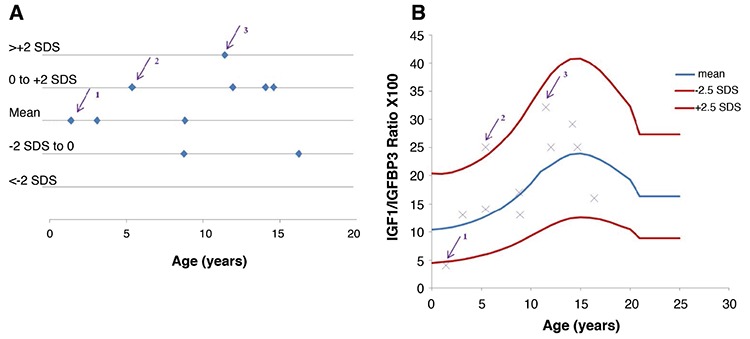
Distribution of IGF1 (A) and IGF1/IGFBP3 molar ratio (B), in children with Prader-Willi syndrome (PWS) during growth hormone (GH) treatment. In the PWS group, three children had discrepancies between serum IGF1 levels [expressed in standard deviation scores (SDS)] and IGF1/IGFBP3 molar ratio. 1^st^ case (arrow 1): 1.4-year-old child with PWS and an IGF1 concentration in the normal range (expressed in SDS) but a low molar ratio (due to very high IGFBP3 concentration >+2 SDS). 2^nd^ case (arrow 2): 5.4-year-old child with PWS and an IGF1 concentration in the normal range (expressed in SDS) but a high IGF1/IGFBP3 molar ratio (due to low IGFBP3 concentration). 3^rd^ case (arrow 3): 11.4-year-old child with a high IGF1 concentration (expressed in SDS) and an IGF1/IGFBP3 molar ratio in the reference range (IGFBP3 concentration towards the upper end of the reference range)

In the group of treated children with PWS, we identified one case in which serum IGF1 concentration was normal but the IGF1/IGFBP3 molar ratio was low due to a high IGFBP3 concentration (+2 SDS), one case in which serum IGF1 concentration was normal but the molar ratio was high due to a very low IGFBP3 concentration (<-2 SDS) and one case in which serum IGF1 concentration was high but the molar ratio was low, due to a high IGFBP3 concentration (Figure 4).

We then assessed the clinical, biochemical and metabolic characteristics of children on rGH with IGF1/IGFBP3 molar ratios inappropriate for age, either higher (>+2SDS) or lower (<-2 SDS) than the normal range (Table 2).

The three study groups were heterogeneous, differing significantly in terms of age and IGF/IGFBP3 molar ratio increases with age. We also noted that both the doses of rGH administered and insulinemia were significantly higher in children with a high molar ratio than in those with a low or normal molar ratio (Table 2). Moreover there was a positive correlation between individual values of insulinemia and the individual values of the whole ratio (p=0.01).

For the other metabolic parameters considered (fasting glycemia, lipid status, HbA1c), we found no significant differences between children with low, normal and high IGF1/IGFBP3 molar ratios (Table 2).

## Discussion

During rGH treatment, increases in the serum concentrations of IGF1 and IGFBP3 are expected because these factors are known to be GH-dependent and to have low basal levels in patients with GHD (13,14).

rGH modifies the distribution of circulating IGF1 between ternary complexes (IGF1/IGFBP3/ALS), binary complexes (IGF1/IGFBP3) and free forms (13). It simultaneously stimulates the production of all three forms, but this effect is stronger for ternary and binary complexes than for free forms (13). The concentration of the bioactive free form of IGF1 does not increase on treatment, even if the total amount of IGF1 increases (13). Thus, rGH increases the levels of IGF1 and IGFBP3 in a heterogeneous, dose-dependent (5,15) manner that differs between individuals (12,13).

In this context, IGFBP3 has been reported to be less sensitive to rGH than IGF1 in adults, with the increase in the concentrations of the former responding less quickly than the latter following rGH therapy (15). This difference results from the hepatic synthesis of IGFBP3s being under the control not only of GH, but also of IGF1s (15). Ranke et al (5) showed that, on rGH treatment, the IGF1-dependent increase in IGFBP3 concentration occurs in two phases: an initial linear phase, followed by a saturation phase in which IGFBP3 concentrations reach a plateau, despite further increases in IGF1 concentration. This would account for the increase in IGF1/IGFBP3 ratio at high doses of rGH. This phenomenon also highlights inequalities in the ability of the liver to synthesize IGF1 (synthesized by hepatocytes) and IGFBP3 (synthesized by Kupffer cells) (16). Thus, at high doses of rGH, serum IGF1 concentration may increase more strongly and more rapidly than IGFBP3 concentration, resulting in an increase in tissue-bioavailable IGF1 concentration (15).

Serum IGF1 and IGFBP3 disorders are known to be associated with a shorter lifespan in humans (17) due to the associated high risk of cardiovascular (18,19,20) and neoplastic comorbidity (21). Indeed, high concentrations of IGF1, a mitogen and anti-apoptotic hormone, have been implicated in the occurrence of various types of cancer (22,23), particularly when associated with low levels of IGFBP3 (24,25,26). In contrast, high serum IGFBP3 concentrations appear to have protective effects against cancer (6) but have been shown to be associated with diabetes, high triglyceride levels and hypertension, whereas low IGFBP3 levels are associated with a large waist circumference and low levels of HDL cholesterol (27).

These findings reflect the U-shaped correlation curve obtained for the relationship between serum IGF1 and IGFBP3 concentrations initially described by the histograms of Juul et al (28) and subsequently confirmed by the “quartiles” of Park and Cohen (29). Indeed, these representations of the risk of cardiovascular ischemia reported by Juul et al (28) and of cardiovascular and neoplastic risks reported by Park and Cohen (29), highlight the need to take both IGF1 and IGFBP3 into account, because these two parameters have “opposite” biological actions (28) and a dynamic “yin-yang” relationship (29).

We investigated the potential utility of the IGF1/IGFBP3 molar ratio for use in the adaptation of rGH treatment in three different etiological groups (children SGA, or with GHD or PWS). Indeed, SGA children are characterized by “GH resistance”, resulting in a need for higher rGH doses than are used in other children matched for age, sex and puberty stage (5,30). In children, rGH dose is generally adjusted progressively, on a case-by-case basis (31). There are currently no clear guidelines on the mode of therapeutic adaptation according to the results obtained (12,29). This complicates therapeutic management, because it is considered important to keep IGF1 concentration below +2 SDS, because of the increased risk of cardiovascular diseases and neoplasm (31).

By contrast, almost all PWS children have a somatotropic deficit (32,33), justifying the systematic (34) early initiation of rGH treatment, within the first year of life (32,33,35). Unlike SGA children, children with PWS are particularly “rGH-sensitive” and have very high serum IGF1 concentrations (towards and above the upper limit of the normal range) (33,34,36).

For the three pathological entities studied (GHD, SGA and PWS), we found that the determination of IGF1/IGFBP3 molar ratio was a useful additional tool for the adaptation of rGH treatment with a view to improving safety.

The safety of rGH treatment is generally ensured by monitoring to keep serum IGF1 concentration at values below the +2 SDS threshold for age and pubertal stage (12). Our results indicate that there may be discrepancies between total IGF1 concentration and IGF1/IGFBP3 molar ratio with a potential impact on the safety of rGH treatment. The use of this ratio made it possible to optimize rGH administration so as to minimize the risk of adverse effects, particular those of a metabolic, cardiovascular or neoplastic nature (17,28,37).

Calculation of the IGF1/IGFBP3 molar ratio is, thus, a potentially useful additional tool because this ratio does not necessarily vary with increases in IGF1 concentration and it takes into account the variation of serum IGFBP3 levels (12).

We were able to determine approximate values for IGF1/IGFBP3 molar ratio before and after the initiation of rGH treatment in children SGA or with GHD or PWS. We found that the mean ratio increased after the initiation of rGH treatment in all groups, but that it remained within the reference range for age and pubertal stage in most children. Our values were consistent with those reported in previous studies. Romer et al (38) reported a low ratio in patients with GHD (0.13±0.07) before rGH, with a sharp increase to more than 0.32±0.07 after treatment initiation. Our results are also consistent with those of Cabrol et al (12), who reported a mean molar ratio before treatment of 0.14 (range: 0.10 to 0.27) in children SGA, increasing to 0.19 (range: 0.15 to 0.23) after treatment.

Finally, on rGH treatment at the doses currently recommended, the IGF1/IGFBP3 molar ratio remained within the normal range for age and sex, even during puberty. We also found that, in children treated with rGH, significant increases in serum IGF1 levels were sometimes associated with IGF1/IGFBP3 molar ratios within the normal range for age and pubertal stage. This suggests that, even in cases of high IGF1 concentration, the action of this molecule is counteracted by an adaptation of IGFBP3 levels, decreasing IGF1 bioavailability.

### Study Limitations

This study included a small number of subjects and our results therefore require confirmation in a larger cohort.

## Conclusion

We consider IGF1/IGFBP3 molar ratio to be a useful additional parameter for assessments of treatment safety and for the adjustment of rGH treatment. The goal would be to maintain this ratio within the normal reference range for age and pubertal stage.

## Figures and Tables

**Table 1A:**
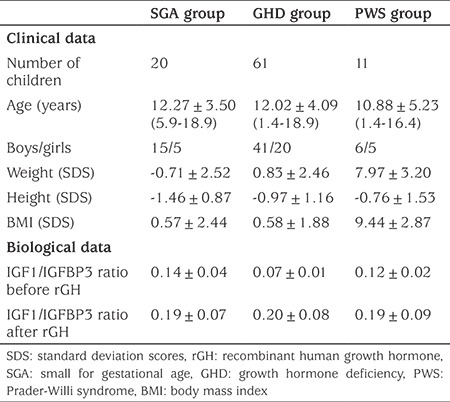
A) Clinical and hormonal characteristics of the children

**Table 1B:**
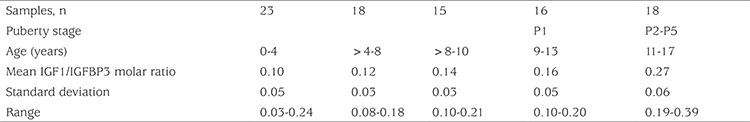
B) Change in IGF1/IGFBP3 molar ratio in control children by age and pubertal stage

**Table 2:**
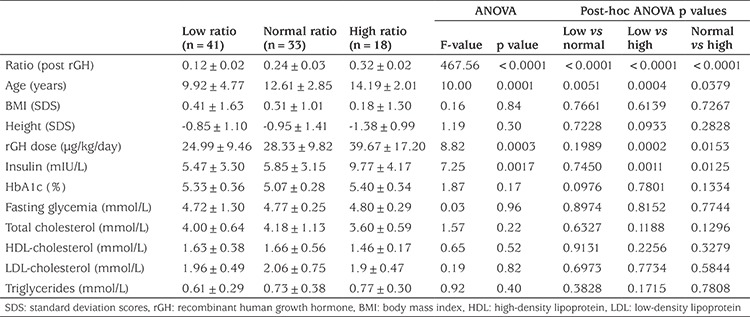
Clinical and metabolic characteristics of children on recombinant human growth hormone with molar ratios higher or lower than the normal range
